# Can endogenous lipid molecules serve as predictors and prognostic markers of coronary heart disease?

**DOI:** 10.1186/1476-511X-7-19

**Published:** 2008-05-20

**Authors:** Undurti N Das

**Affiliations:** 1 UND Life Sciences, 13800 Fairhill Road, #321, Shaker Heights, OH 44120, USA

## Abstract

Dyslipidemia, and inflammatory markers: high-sensitivity C-reactive protein (hs-CRP), myeloperoxidase (MPO), lipoprotein associated phospholipase A_2_(Lp-PLA_2_), and lipid peroxides (LP) are insufficient to predict the onset, extent, and prognosis of CHD. Lipoxins (LXs), resolvins, and protectins are derived from ω-3 fatty acids: eicosapentaenoic acid (EPA) and docosahexaenoic acid (DHA), and ω-6 arachidonic acid in the presence of aspirin; whereas nitrolipids are formed due to the interaction between polyunsaturated fatty acids and nitric oxide (NO). LXs, resolvins, protectins, and nitrolipids are endogenous anti-inflammatory lipid molecules that inhibit production of interleukin-6 (IL-6) and tumor necrosis factor- α (TNF-α), suppress free radical generation, enhance NO generation; and accelerate tissue repair. Thus, beneficial actions of EPA/DHA and aspirin in CHD could be attributed to the formation of LXs, resolvins, protectins, and nitrolipids and suggest that their plasma levels aid in the prediction and prognosis of CHD.

## Introduction

An increase in the plasma concentrations of high-sensitive C-reactive protein (hs-CRP), tumor necrosis factor-α (TNF-α), and interleukin-6 (IL-6) occurs in coronary heart disease (CHD) suggesting that it is a low-grade systemic inflammatory condition [1]. Myeloperoxidase (MPO), an abundant leukocyte enzyme, and lipoprotein-associated phospholipase A_2 _(Lp-PLA_2_), produced by macrophages, independently predict the early risk of adverse cardiac events [2,3]. Despite these advances, it is not clear which of these indices are better suited to predict CHD, its severity, and consequent complications.

Although, dyslipidemia, diabetes mellitus, hypertension, and obesity are important risk factors for CHD, there are a significant number of subjects who develop CHD without these risk factors. We and others observed that in CHD, hypertension, diabetes mellitus, hyperlipidemias, and obesity, EFA (essential fatty acid) metabolism is abnormal such that plasma phospholipid concentrations of arachidonic acid (AA), eicosapentaenoic acid (EPA), and docosahexaenoic acid (DHA) are low [4-11]. Despite the fact that deficiency of polyunsaturated fatty acids (PUFAs) is present in CHD, hypertension, diabetes, hyperlipidemias, and obesity and increased intake of PUFAs are of benefit in these diseases [4,7,9-11], exact mechanism(s) of their protective action remained unexplained.

### Metabolism of essential fatty acids and its relevance to CHD

**Figure 1:**
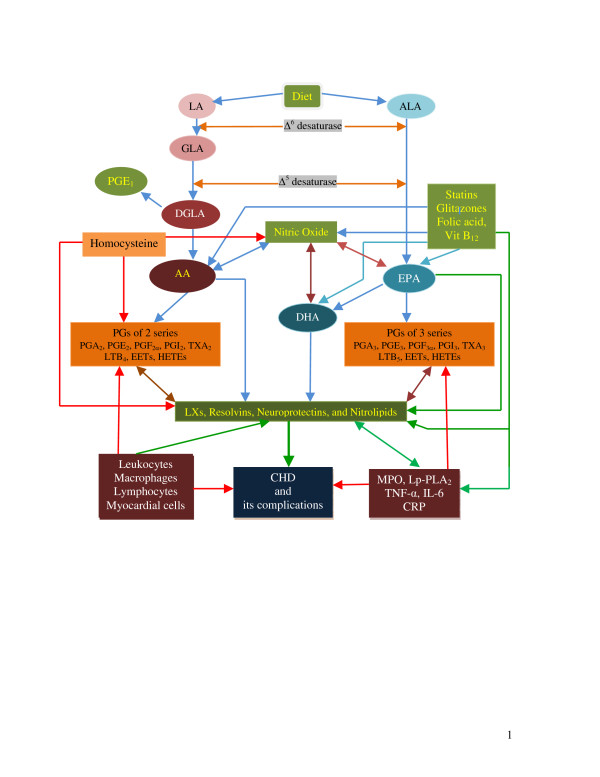
Scheme showing the metabolism of essential fatty acids. Red line indicates initiation and/or progression of disease. Green line indicates protection from disease and better prognosis. Double arrow indicates feedback regulation that could be both positive and negative. Leukocytes, macrophages, lymphocytes, and myocardial cells produce various PGs, LTs, TXs, cytokines, MPO, and other pro-inflammatory molecules that participate in the pathogenesis of CHD. Leukocytes, macrophages, lymphocytes, myocardial cells also produce lipoxins, resolvins, NPD_1_, and nitrolipids that have cardioprotective action and enhance healing and prevent or arrest atherosclerosis. Hence, measuring these cardiotoxic and cardioprotective molecules may help in the prediction and prognosis of CHD, and response to therapy.

Essential fatty acids (EFAs) are important constituents of all cell membranes including endothelial and myocardial cells and determine and influence the behaviour of membrane-bound enzymes and receptors by altering membrane fluidity. EFAs are essential, not synthesized in the body; and hence, have to be obtained in diet [4]. The two EFAs are ω-6 *cis*-linoleic acid (LA, 18:2) and the ω-3 α-linolenic acid (ALA, 18:3). AA formed from LA is the precursor of 2 series of PGs, thromboxanes (TXs) and 4 series of leukotrienes (LTs); whereas ALA is converted to EPA that gives rise to 3 series of PGs, TXs and 5 series LTs (see Figure 1). LA, γ-linolenic acid (GLA), dihomo-GLA (DGLA), AA, ALA, EPA and docosahexaenoic acid (DHA, 22:6, ω-3) are all PUFAs, but only LA and ALA are EFAs. AA and EPA and their respective PGs, TXs, and LTs play a significant role in atherosclerosis, CHD, bronchial asthma, inflammatory bowel disease, and other inflammatory conditions [4].

AA, EPA, and DHA give rise to LXs, resolvins, and protectins. Aspirin converts AA, EPA and DHA to form aspirin-triggered 15 epimer LXs (ATLs) that inhibit local inflammation on the vessel wall by regulating the motility of leukocytes, eosinophils, and monocytes [4,12-14]. COX-2 enzyme is essential for the formation of LXs, whose deficiency leads to an interaction between leukocytes (PMNs) and endothelial cells that result in endothelial damage, initiation and progression of atherosclerosis, thrombus formation and CHD.

EPA can be converted to 18R-HEPE (18R- hydroxy-eicosapentaenoic acid), 18-HEPE, and 15R-HEPE. Activated human PMNs, in turn, convert 18R-HEPE to 5,12,18R-triHEPE and 15R-HEPE to 15-epi-LXA_5 _by 5-lipoxygenase. Both 18R-HEPE and 5,12,18R-triHEPE inhibited LTB_4_-stimulated PMN transendothelial migration similar to 15-epiLXA_4_. 5,12,18R-triHEPE competed with LTB_4 _for its receptors and inhibited PMN infiltration, and thus, 5,12,18R-triHEPE suppresses LT-mediated responses when present at the sites of inflammation [4,12-15].

Similarly, DHA is transformed enzymatically to 17R series of hydroxy DHAs (HDHAs) that, in turn, is converted by PMNs to di- and tri-hydroxy containing docosanoids [4,16]. Similar small molecular weight compounds (similar to HDHAs) are generated from AA and EPA. Thus, 15R-hydroxy containing compounds are formed from AA, 18R series from EPA, and 17R-hydroxy series from DHA that have potent anti-inflammatory actions and induce resolution of the inflammatory process and are called "resolvins" (see Figure 1). Resolvins inhibited cytokine generation, leukocyte recruitment, leukocyte diapedesis, and exudate formation. AA, EPA, and DHA-derived resolvins inhibited brain ischemia-reperfusion injury. 10,17S-dihydroxydocosatriene derived from DHA termed as neuroprotectin D1 (NPD1) reduced infiltration of PMNs, showed anti-inflammatory and neuroprotective properties [4,17], and inhibited oxidative stress-induced apoptosis of human retinal pigment epithelial cells [18]. Both LXs and NPD1 enhanced wound healing [19], and promoted brain cell survival [20,21]. Thus, lipoxins, resolvins, and protectins derived from AA, EPA, and DHA have cardio-protective, neuroprotective, and other cytoprotective actions. It is noteworthy that leukocytes, macrophages, endothelial cells, and myocardial cells have the complete enzyme system for the conversion of PUFAs to PGs, TXs, LTs, LXs, resolvins, protectins and nitrolipids. Based on these findings, I propose that failure to produce adequate amounts of LXs, resolvins, NPD1 (and other protectins), and nitrolipids or interference with their action could lead to initiation and persistence of inflammation and tissue damage including CHD.

### Nitrolipids and CHD

NO reacts with PUFAs to yield their respective nitroalkene derivatives, termed as nitrolipids, produce vascular relaxation, inhibit neutrophil degranulation and superoxide formation, and inhibit platelet activation [22-24]. Nitrolipids have endogenous PPAR-γ ligand activity and release NO [24]. Thus, nitrolipids prevent platelet aggregation, thrombus formation, atherosclerosis, and suppress inflammation. Thus PUFAs not only form precursors to various eicosanoids, resolvins, LXs, and NPD1 but also react with NO to form nitrolipids that prevent platelet aggregation and thrombus formation and hence, arrest of atherosclerosis. Nitrolipids also have anti-inflammatory actions, and so, their deficiency can cause CHD.

### Pro- and anti-inflammatory molecules in CHD

Coronary heart disease (CHD) is a low-grade systemic inflammatory condition since these subjects have enhanced plasma levels of reactive oxygen species (ROS), CRP, IL-6, and TNF-α and low circulating levels of NO and various PUFAs [1-6]. Increased ROS decreases anti-oxidant content of the cells/tissues. This leads to an imbalance between the pro- and anti-oxidant molecules that favor tissue damage in CHD [25]. Hs-CRP, LP-LPA_2_, ROS, and MPO have cytotoxic actions, whereas LXs, resolvins, protectins, and nitrolipids show cytoprotective and cardioprotective actions, in view of their ability to inhibit production of IL-6 and TNF-α, suppress free radical generation, and enhance tissue repair [4,12-24,26-28]. Hence, an increase in hs-CRP, LP-LPA_2_, ROS, and MPO and a decrease in LXs, resolvins, protectins, and nitrolipids or both not only predisposes to CHD but also indicates poor prognosis. When EPA, DHA, AA, and aspirin are given together in adequate amounts, LXs, resolvins, protectins, and nitrolipids are formed that prevent myocardial damage. This explains the beneficial actions of EPA/DHA/AA and aspirin in both primary and secondary prevention of CHD [4,26-28].

This implies that deficiency of PUFAs seen in CHD and other conditions leads to reduced formation of NO, LXs, resolvins, protectins, and nitrolipids that would initiate atherosclerosis and CHD and/or worsen an existing disease. It is predicted that plasma levels of hs-CRP, ROS, MPO, Lp-PLA_2 _and LP will be increased whereas those of LXs, resolvins, NPD_1 _and nitrolipids will be decreased in patients with CHD. Furthermore, a balance between these pro- and anti-inflammatory molecules may aid in predicting prognosis of CHD. For instance, subjects with low levels of LXs, resolvins, NPD_1_, and nitrolipids may have poor outcome and have higher incidence of cardiac failure, arrhythmias, and recurrence of myocardial infarction. Statins and thiazolidinediones enhance the formation of myocardial 15-epi-LXA_4 _and EFAs mediate some of their actions that could explain the ability of statins and glitazones to prevent CHD [29-31]. This implies that those who have low levels of EFAs and their metabolites could be resistant to the beneficial action of statins and glitazones; and such subjects will be benefited from supplementation of AA/EPA/DHA.

### Hypothesis: EFAs/PUFAs and their metabolites are low in CHD

One of the risk factors for CHD is elevated levels of homocysteine. Southeast Asians especially, Indians (people from Indian subcontinent) are at high risk of CHD [32]. The increased incidence of CHD in Indians has been attributed to their proatherogenic diet, lack of exercise, smoking, high levels of homocysteine and LP(a) lipoprotein, endothelial dysfunction and enhanced plaque and low-grade systemic inflammation [32]. They also have increased prevalence of metabolic syndrome X including insulin resistance, hypertension, abdominal obesity, and dyslipidemia, and type 2 diabetes mellitus, factors that could explain the high incidence of CHD. These factors coupled with the observation that only limited number of patients with CHD receive thrombolytic therapy (36.8%), antiplatelet drugs (~98%), β-blockers (59.3%), lipid-lowering drugs (52%), ACE inhibitors/angiotensin-II receptor blockers (56.8%), anticoagulants (81.3%), percutaneous coronary intervention (7.5%), coronary artery bypass graft (2.9%) and that the incidence of death and complications are more common in Indians [32,33] suggests that as a race Indians are the most vulnerable population for cardiovascular diseases. It is estimated that by 2010, 60% of the world's heart disease is expected to occur in India [34] and are likely to suffer from CHD at an earlier age than do people in developed countries [35]. Unfortunately, all the studies performed in Indians, residing both in India and the developed countries; concentrated on the incidence of the disease and the prevalence of risk factors but none have looked at the pathophysiology of the disease. In a previous study, I showed that even healthy Indians have lower concentrations of various PUFAs compared to Canadians and Americans (USA) [36]. This suggests that as a race Indians have low activity of Δ^6 ^and Δ^5 ^desaturases that is responsible for decreased concentrations of various PUFAs in their plasma and tissues as a result of which the formation of PGE_1_, PGI_2_, PGI_3_, lipoxins, resolvins and protectins will be low that may render them more susceptible to develop low-grade systemic inflammatory conditions including CHD [36-41]. This is further supported by the observation that Indians and Europeans with CHD have low levels of PUFAs in their plasma phospholipid fraction [6,37,38]. Furthermore, low activity of Δ^6 ^and Δ^5 ^desaturases could lead to the initiation and progression of insulin resistance and atherosclerosis in Indians as previously proposed [42,43]. These evidences suggest that Indians are at high risk of developing CHD and its associated death and complications [32,33] partly due to a defect in the activity of Δ^6 ^and Δ^5 ^desaturases that result in low plasma and tissue concentrations of PUFAs and their products: PGE_1_, PGI_2_, PGI_3_, lipoxins, resolvins, protectins, and nitrolipids [36-41]. PUFAs and their products prevent platelet aggregation, lower blood pressure, reduce LDL-C, ameliorate the adverse actions of homocysteine, inhibit ACE (angiotensin converting enzyme) and HMG-CoA enzyme activities and thus, may function as endogenous "polypill" [44]. These evidences suggest that PUFAs of both ω-3 and ω-6 series can be given to high risk subjects to prevent CHD and other cardiovascular diseases [44]. It is likely that subjects with low levels of various PUFAs and their products: PGE_1_, PGI_2_, PGI_3_, lipoxins, resolvins, protectins, and nitrolipids are not only vulnerable to develop CHD but also have poor outcome and higher incidence of cardiac failure, arrhythmias, and recurrence of myocardial infarction (Figure 1) since these endogenous lipid molecules have anti-arrhythmic action, enhance wound healing and suppress inflammation [45-49], though some studies disputed the anti-arrhythmic action of PUFAs [50,51]. In this context, it is interesting to note that folic acid not only reduces plasma homocysteine levels but also augments the metabolism of essential fatty acids such that plasma and tissue levels of various PUFAs are enhanced (reviewed in [44,52-55]). It is possible that some subjects may have normal concentrations of EFAs/PUFAs in their plasma and tissues but the formation of lipoxins, resolvins, protectins, and nitrolipids may be defective. Recently it was noted that the gene that encodes the 5-lipoxygenase activating protein (FLAP) and its risk variant results in an almost 2-fold increased risk of CHD by leading to the production of leukotriene B_4 _(LTB_4_), a potent chemokine mediators of arterial inflammation [56-58]. This is supported by the observation that patients with CHD produce more LTB_4 _than controls, suggesting that LTB pathway is upregulated in CHD [56]. It is interesting to note that lipoxins are potent inhibitors of LTB_4 _and leukotrienes [59].

Thus, it is proposed that subjects at high risk for CHD have increased hs-CRP, MPO, Lp-PLA_2_, LP, IL-6, TNF-α, LTB_4 _and low levels of various PUFAs, LXs, resolvins, protectins, and nitrolipids, and the balance between these pro-inflammatory and anti-inflammatory molecules may also aid in the prediction and prognosis of CHD.

If this proposal is true, it implies that PUFAs are not only useful in the prevention of CHD but development of stable and synthetic analogues of LXs, resolvins, NPD_1_, and nitrolipids may form a new approach in the management of cardiovascular diseases.
